# Compartment-Specific Changes in SESN2 and NRF2 Expression Following Narrowband UVB Therapy in Early-Stage Mycosis Fungoides

**DOI:** 10.3390/jcm15218225

**Published:** 2026-10-22

**Authors:** İlkay Özer, Hanife Uçgun Demirtaş, Fatih Kılıç, Naile Kökbudak, Selami Aykut Temiz, Recep Dursun, Munise Daye

**Affiliations:** 1 Department of Dermatology and Venereology, Faculty of Medicine, Necmettin Erbakan University, Konya 42060, Türkiyesatemiz@erbakan.edu.tr (S.A.T.); rdursun@erbakan.edu.tr (R.D.); mdaye@erbakan.edu.tr (M.D.); 2 Department of Dermatology, Hendek State Hospital, Sakarya 54300, Türkiye; 3 Department of Pathology, Faculty of Medicine, Necmettin Erbakan University, Konya 42060, Türkiye

**Keywords:** mycosis fungoides, narrowband UVB, NRF2, oxidative stress, phototherapy, tumor microenvironment, SESN2

## Abstract

**Background and Objectives:** Mycosis fungoides (MF) is the most common primary cutaneous T-cell lymphoma, and oxidative stress within the tumor microenvironment may contribute to its pathogenesis and treatment response. Sestrin 2 (SESN2) and nuclear factor erythroid 2-related factor 2 (NRF2) are key regulators of cellular responses to oxidative stress. This study aimed to evaluate changes in epidermal and dermal SESN2 and NRF2 expression following narrowband ultraviolet B (NB-UVB) therapy in patients with early-stage MF. **Materials and Methods:** This retrospective observational study included 29 patients with stage IA–IIA MF treated with NB-UVB phototherapy. Paired skin biopsies obtained from the same anatomical site before treatment and after histopathological remission were evaluated immunohistochemically for epidermal and dermal SESN2 and NRF2 expression. Staining intensity, positivity, and H-scores were assessed semiquantitatively. **Results:** Following NB-UVB therapy, dermal NRF2 staining intensity, positivity, and H-score decreased significantly (all unadjusted *p* ≤ 0.002), and these differences remained significant after adjustment for multiple comparisons. Epidermal SESN2 positivity and H-score increased in the unadjusted analyses (*p* = 0.047 and *p* = 0.030, respectively), but these differences did not remain statistically significant after adjustment. No significant changes were observed in dermal SESN2 or epidermal NRF2 expression. Changes in SESN2 and NRF2 H-scores were not significantly associated with cumulative NB-UVB dose. No significant associations were identified between SESN2 or NRF2 expression parameters and time to histopathological remission. **Conclusions:** Histopathological remission following NB-UVB therapy in early-stage MF was associated with significantly reduced dermal NRF2 expression after adjustment for multiple comparisons, whereas no statistically significant changes were observed in epidermal NRF2 or epidermal and dermal SESN2 expression. Further studies are required to determine whether the reduction in dermal NRF2 reflects changes in the cellular composition of the infiltrate, altered expression within individual cells, or both.

## 1. Introduction

Mycosis fungoides (MF) is the most common primary cutaneous T-cell lymphoma. Although genetic, antigenic, immunological, molecular, and environmental factors are thought to contribute to its development by inducing chronic antigenic stimulation and uncontrolled clonal proliferation of memory helper T cells, its etiopathogenesis remains incompletely understood. In the early stages of the disease, which generally follows a chronic and indolent course, skin-directed therapies, including topical corticosteroids, topical retinoids, narrowband ultraviolet B (NB-UVB), and psoralen plus ultraviolet A (PUVA), are commonly used [1]. In MF lesions, neoplastic T cells generally constitute a minority of the cellular population within a complex tumor microenvironment comprising reactive T cells, macrophages, fibroblasts, dendritic cells, mast cells, and myeloid-derived suppressor cells [2]. In addition to neoplastic cells themselves, the tumor microenvironment and the reciprocal interactions between neoplastic cells and their surrounding microenvironment, together with the mediators produced within this milieu, play important roles in treatment response and disease progression [3]. A microenvironment characterized by hypoxia and oxidative stress is observed in many malignancies. These conditions can induce structural and functional alterations in components of the tumor microenvironment and influence the biological behavior of malignant cells [4]. Increased levels of reactive oxygen species (ROS) may contribute to multiple stages of malignant progression, from tumor initiation to metastatic dissemination. Conversely, depending on the characteristics of the tumor microenvironment, ROS may also suppress tumorigenesis [5]. Therapeutic approaches that induce oxidative stress by increasing ROS levels are emerging as promising strategies for the treatment of cutaneous T-cell lymphomas [6].

Sestrin 2 (SESN2) and nuclear factor erythroid 2-related factor 2 (NRF2) are important components of cellular defense systems that protect cells against hypoxia, oxidative stress, inflammation, and metabolic stress [7]. SESN2 is a stress-responsive protein whose expression increases under various cellular stress conditions, including hypoxia, oxidative stress, and DNA damage. It protects cells against stress-induced injury and contributes to the maintenance of cellular homeostasis [8]. NRF2 is also a key component of the cellular defense response to oxidative stress and promotes cell survival under stressful conditions. NRF2 activity increases in response to oxidative stress, whereas under normal or unstressed conditions, NRF2 is rapidly degraded in the cytoplasm, thereby maintaining low basal levels [9]. Phototherapy, including NB-UVB, is widely used to treat various dermatological conditions and can both induce oxidative stress and activate cellular antioxidant defense mechanisms. Accordingly, markers of oxidative stress have been proposed as potentially useful tools for monitoring disease activity and treatment response in dermatological disorders [10].

SESN2 and NRF2 were selected as complementary regulators of cellular stress responses with potential relevance to NB-UVB-treated MF. Experimental studies linking SESN2 to UVB-induced stress responses in skin cells provide a photobiological rationale for its investigation, whereas evidence implicating NRF2 in lymphoma biology and redox dysregulation in CTCL provides a disease-related rationale [11,12]. Separate evaluation of epidermal and dermal SESN2 and NRF2 immunoreactivity may help characterize compartment-specific staining patterns and provide a framework for interpreting changes associated with treatment and remission.

Although oxidative stress has been extensively investigated in various malignancies, its relationship with MF and treatment-associated changes in redox-regulatory proteins remains incompletely understood. This study therefore aimed to compare epidermal and dermal SESN2 and NRF2 expression before NB-UVB phototherapy and after clinical and histopathological remission in patients with early-stage MF using immunohistochemistry. We hypothesized that remission following NB-UVB therapy would be associated with compartment-specific changes in SESN2 and NRF2 expression, potentially reflecting changes in cellular stress responses and the composition of the tumor microenvironment.

## 2. Materials and Methods

This retrospective observational study was conducted following approval by the Ethics Committee of Necmettin Erbakan University (protocol code 2025/5744, 25 April 2025). Patients who were followed at the Department of Dermatology and Venereology, Necmettin Erbakan University, between June 2016 and January 2025, had a clinical and histopathological diagnosis of early-stage mycosis fungoides (stage IA–IIA), and received NB-UVB phototherapy three times per week were included in the study. Patients who were pregnant or breastfeeding, those younger than 18 years, those in whom pre-treatment and histopathological remission biopsies could not be confirmed from the same anatomical site, those without sufficient paraffin-embedded tissue for additional immunohistochemical staining, and those who received concomitant therapies in combination with NB-UVB, including topical corticosteroids, topical retinoids, systemic interferon, methotrexate, or systemic bexarotene, were excluded.

Before initiation of phototherapy, the minimal erythema dose (MED) was determined for each patient, and treatment was initiated at 70% of the MED. NB-UVB phototherapy was administered three times per week. At each session, patients were evaluated for the presence of erythema, and subsequent doses were increased by 10–20% of the previous dose according to the erythema response. The corresponding anatomical locations of tissues from patients with mycosis fungoides who had achieved histopathological remission were verified using photographs taken routinely before each biopsy and stored in the patients’ medical records. Lesional skin specimens obtained at the time of histopathological diagnosis and post-treatment specimens obtained from the same anatomical site after histopathological remission were subjected to immunohistochemical staining for SESN2 and NRF2. For each patient, the interval between the date of diagnosis and the date on which histopathological remission was first documented was calculated in weeks and defined as the “time to histopathological remission”. Immunohistochemically stained sections were evaluated semi-quantitatively under a light microscope (Olympus BX51; Olympus Corporation, Tokyo, Japan) by an independent pathologist blinded to the patients’ previous pathological findings, demographic characteristics, and the timing of the specimen (pre- or post-treatment). For each specimen, randomly selected high-power fields (×400) containing at least 200 cells were examined in both the epidermis and the dermis. Complete histopathological response, defined by the disappearance of epidermotropism, absence of atypical lymphocytic infiltration, and absence of Pautrier microabscesses, was used as the criterion for histopathological remission. SESN2 and NRF2 immunostaining was evaluated semi-quantitatively, with the epidermis and dermis assessed separately in accordance with the histological architecture of the affected skin and the specific cellular staining patterns of each marker. For the evaluation of SESN2 and NRF2 immunostaining intensity, staining was scored as follows: 0, no staining; 1, weak staining; 2, moderate staining; and 3, strong staining. For the assessment of the percentage of stained cells, the absence of staining was assigned a value of 0. Epidermal and dermal staining was evaluated separately. Staining intensity was graded as 0 (negative), 1+ (weak), 2+ (moderate), or 3+ (strong). The percentage of cells at each staining intensity was determined relative to the total number of cells evaluated within the corresponding compartment, with the percentages across all four categories summing to 100%. The H-score was calculated as follows: H-score = (1 × percentage of weakly stained cells) + (2 × percentage of moderately stained cells) + (3 × percentage of strongly stained cells), yielding a score ranging from 0 to 300. SESN2 and NRF2 immunoreactivity was evaluated separately in the epidermal and dermal compartments. The cellular distribution of positive staining was described qualitatively on the basis of morphological features.

## 3. Immunohistochemical Staining

Sections 3 μm in thickness were obtained from paraffin-embedded tissue blocks and mounted on glass slides. The sections were incubated at 60 °C for 1 h and subsequently deparaffinized in xylene for three 5-min cycles. The slides were then rehydrated through a graded series of decreasing ethanol concentrations (100%, 96%, 80%, and 70%) and rinsed twice in distilled water for 1 min each to remove residual alcohol. For antigen retrieval, 1:10 diluted EDTA buffer (pH 8; AP-9004-1000, Thermo Scientific, Fremont, CA, USA) was applied using a PT Module (A80400012, Lab Vision, Fremont, CA, USA). The slides were then placed with coverplates into the racks of the immunohistochemistry staining system and washed with phosphate-buffered saline (PBS) for 5 min. Endogenous peroxidase activity was blocked by incubation with 3% hydrogen peroxide (TA-125-HP, Thermo Scientific) for 10 min, followed by washing with PBS. Protein blocking solution (TA-125-PBQ, Thermo Scientific) was then applied for 10 min. The sections were incubated for 2 h with NRF2 antibody (orb128433, Biorbyt, Cambridge, UK; dilution 1:75) and SESN2 antibody (66297-1-Ig, Proteintech, Rosemont, IL, USA; dilution 1:100). After washing with PBS for 5 min, Amplifier Quanto (TL-125-QPB, Thermo Scientific) was applied for 20 min, followed by HRP Polymer Quanto (TL-125-QPH, Thermo Scientific) for 30 min. PBS washing was performed between each step. Positive immunoreactivity was visualized using DAB Chromogen (TA-125-HA, Thermo Scientific). The sections were counterstained with hematoxylin (HHS32, Sigma St. Louis, MO, USA) for 1 min and subsequently washed twice in distilled water for 1 min each. The slides were then dehydrated through a graded series of increasing ethanol concentrations (70%, 80%, 96%, and 100%), immersed in xylene twice for 1 min each, and mounted with Entellan. Immunohistochemical staining was performed using a Sequenza Immunostaining Center (73300001, Shandon/Thermo Scientific, Runcorn, UK).

## 4. Statistical Analysis

Paired specimens from individual patients were identified using matched file/case numbers. Demographic and clinical variables, including age, sex, and disease stage; treatment-related parameters, including time to histopathological remission in weeks and cumulative NB-UVB dose (mJ/cm^2^) at the end of treatment; and epidermal and dermal SESN2 and NRF2 expression parameters, including staining intensity score, percentage of positively stained cells, and H-score, were recorded. Descriptive statistics were calculated for all variables. Continuous variables were summarized as mean ± standard deviation and median (minimum–maximum), or median (interquartile range) for skewed distributions, as appropriate. Categorical variables, including sex and disease stage, were expressed as frequencies and percentages. Given the relatively small sample size and the ordinal nature of several outcome variables, particularly staining intensity scores, as well as the non-normal and right-skewed distribution of some continuous variables, such as cumulative NB-UVB dose, a non-parametric approach was adopted for the primary comparisons. Pre-treatment and post-treatment epidermal and dermal SESN2 and NRF2 expression parameters were compared within the same patients using the Wilcoxon signed-rank test for paired samples. All tests were two-sided, and a *p*-value < 0.05 was considered statistically significant. A secondary analysis was performed to determine whether the magnitude of the observed pre-to-post-treatment changes was influenced by the cumulative NB-UVB dose. For each biomarker, an individual change score (post-treatment value minus pre-treatment value) was calculated, and its association with cumulative NB-UVB dose was assessed using Spearman’s rank correlation coefficient. Pre- and post-treatment values of intensity, percentage of positive cells and H-score were compared separately for each marker and compartment using the Wilcoxon signed-rank test. Effect sizes were calculated as r = |Z|/√*n* and interpreted as small (0.1), medium (0.3) or large (≥0.5). To control the family-wise error rate arising from multiple comparisons, *p* values were adjusted using the Holm step-down method. All statistical analyses were performed using Python version 3 with the SciPy and pandas libraries. Results are presented as median (interquartile range), together with unadjusted and Holm-adjusted *p*-values and effect sizes. Associations between changes in H-scores and cumulative NB-UVB dose were assessed separately using Spearman’s rank correlation. Holm correction was applied across all 12 pre–post comparisons, and these adjusted *p*-values formed the basis for the main interpretation. Additional Holm-adjusted *p*-values were calculated across the four comparisons within each outcome measure.

## 5. Results

Data were obtained from 29 patients with mycosis fungoides who underwent NB-UVB phototherapy and had paired pre-treatment and post-treatment skin biopsies, yielding a total of 58 biopsy specimens. Epidermal and dermal SESN2 and NRF2 expression levels, along with demographic, clinical, and treatment-related parameters, were recorded for all patients. The demographic and clinical characteristics of the study population are summarized in Table 1.

**Figure 1:**
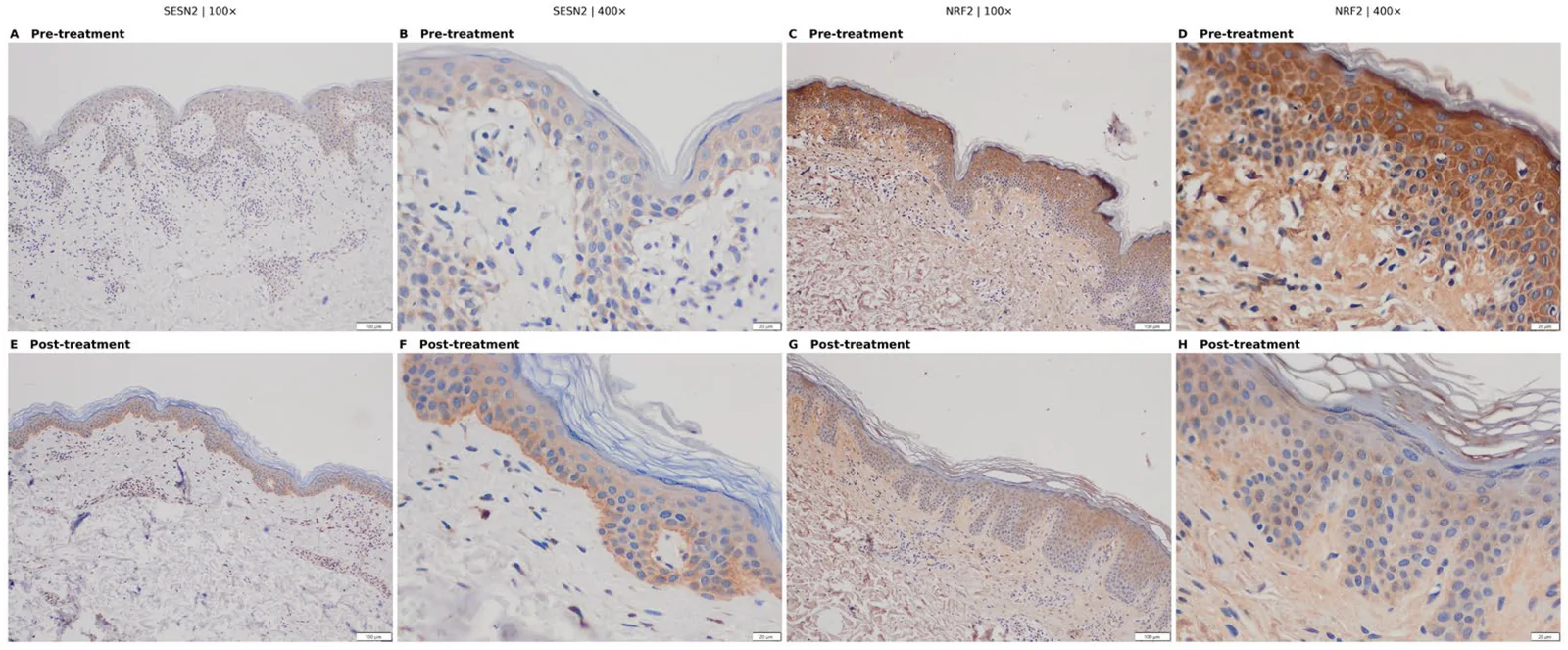
Representative immunohistochemical staining for SESN2 and NRF2 in skin specimens obtained before and after narrowband ultraviolet B (NB-UVB) therapy in patients with early-stage mycosis fungoides. The upper row shows pretreatment specimens (**A**–**D**), and the lower row shows post-treatment specimens (**E**–**H**). SESN2 immunostaining is presented at ×100 (**A**,**E**) and ×400 (**B**,**F**), and NRF2 immunostaining at ×100 (**C**,**G**) and ×400 (**D**,**H**). The images illustrate epidermal keratinocyte staining and dermal immunoreactivity. Scale bars: 100 μm (**A**,**C**,**E**,**G**) and 20 μm (**B**,**D**,**F**,**H**).

In the epidermis, immunoreactivity for both SESN2 and NRF2 was confined to keratinocytes. In the dermis, SESN2 immunoreactivity was observed in lymphocytes, whereas NRF2 immunoreactivity was observed in cells morphologically consistent with fibroblasts, eosinophils, mast cells, lymphocytes, and histiocytes. Cell types were identified on the basis of morphological features. Representative immunohistochemical images illustrating the epidermal and dermal staining patterns of both markers are presented in Figure 1.

In the unadjusted analyses, pre-treatment and post-treatment epidermal and dermal SESN2 and NRF2 expression parameters were compared using the Wilcoxon signed-rank test for paired samples (Table 2). Following NB-UVB phototherapy, epidermal SESN2 positivity and H-score increased significantly compared with pre-treatment values (*p* = 0.047 and *p* = 0.030, respectively). In contrast, dermal NRF2 staining intensity, positivity, and H-score decreased significantly following treatment (all *p* ≤ 0.002). No significant pre- to post-treatment differences were observed in dermal SESN2 or epidermal NRF2 expression parameters (all *p* > 0.05). The observed changes in dermal NRF2 expression remained statistically significant following adjustment for multiple comparisons; however, the increase in epidermal SESN2 expression was no longer statistically significant after adjustment (Table 2).

To determine whether the observed changes were associated with cumulative NB-UVB exposure, the relationship between the magnitude of change in each parameter (post-treatment minus pre-treatment value) and the cumulative NB-UVB dose (mJ/cm^2^) administered by the end of treatment was assessed using Spearman’s rank correlation analysis (Table 3). No statistically significant correlations were observed between cumulative NB-UVB dose and changes in SESN2 or NRF2 H-scores (all *p* > 0.30).

Spearman’s rank correlation analysis was used to assess associations between time to histopathological remission (weeks) and baseline H-scores, post-treatment H-scores, and changes in H-scores (post-treatment minus baseline) for epidermal and dermal SESN2 and NRF2 (Table 4). No statistically significant associations were identified (all *p* ≥ 0.129). Baseline dermal NRF2 H-score showed a non-significant inverse correlation with time to histopathological remission (ρ = −0.29, *p* = 0.129).

## 6. Discussion

The principal finding of this study was that histopathological remission following NB-UVB treatment in early-stage MF was associated with a significant reduction in dermal NRF2 expression after adjustment for multiple comparisons. No statistically significant changes were observed in epidermal NRF2 or epidermal and dermal SESN2 expression after adjustment. These findings provide partial support for the hypothesis of compartment-specific changes associated with remission, with statistically significant changes confined to dermal NRF2 after adjustment for multiple comparisons.

In addition to neoplastic cells, the tumor microenvironment plays an important role in pathways involved in malignancy, and reciprocal interactions between neoplastic T lymphocytes and the tumor microenvironment contribute to treatment response and disease progression [13]. Accordingly, therapeutic strategies targeting alterations within the tumor microenvironment have been proposed as promising approaches for the treatment of MF [14]. Previous studies have demonstrated that the MF microenvironment evolves into a reprogrammed state characterized by pro-inflammatory and immunosuppressive features, hypoxia, and increased oxidative stress, with oxidative stress being associated with disease progression [15,16,17]. Although increased oxidative stress may promote malignant transformation by enhancing genomic instability, malignant cells also require highly efficient antioxidant defense mechanisms for survival. This dependence may render them vulnerable to therapeutic strategies that further increase oxidative stress, thereby highlighting the dual role of oxidative stress in malignancy [18]. Furthermore, oxidative stress may be an important determinant of treatment response in cutaneous T-cell lymphoma. The combination of dimethyl fumarate and 8-methoxypsoralen/UVA has been reported to enhance malignant T-cell death by reducing thioredoxin reductase activity and glutathione levels, thereby increasing reactive oxygen species and activating ferroptotic, necroptotic, and apoptotic pathways in a cell line-dependent manner [19]. PUVA and NB-UVB phototherapy are widely used in the treatment of early-stage MF. Compared with PUVA, NB-UVB has been described as having a relatively more favorable adverse-effect profile in this setting [20]. PUVA and NB-UVB exert their therapeutic effects through distinct mechanisms. In PUVA, psoralen interacts with DNA upon UVA exposure, inducing DNA damage, while ROS contribute to damage to cellular organelles. UVA has also been reported to induce p53-independent apoptosis, with the phototoxic effects preferentially targeting malignant T cells. Although the mechanism of action of NB-UVB in MF remains incompletely understood, its therapeutic effects have been associated with suppression of the antigen-presenting capacity of Langerhans cells, modulation of cytokine profiles, and induction of T-cell apoptosis [21].

SESN2 and NRF2 are two important molecules involved in the cellular response to oxidative stress. SESN2, encoded by the hypoxia-inducible gene 95, is induced under conditions such as DNA damage, oxidative stress, hypoxia, and nutrient deprivation. It contributes to the maintenance of cellular homeostasis through activation of 5′-adenosine monophosphate-activated protein kinase (AMPK) and inhibition of mammalian target of rapamycin (mTOR) signaling and is involved in the maintenance of mitochondrial homeostasis and regulation of autophagy. Although SESN2 participates in processes critical to tumor development and progression, including tumor cell proliferation, apoptosis, differentiation, progression, and treatment response, it may also exert oncogenic functions under certain conditions [22]. Although the relationship between sestrins, oxidative stress, and malignant progression has yielded conflicting findings in the literature, Sestrin expression is reduced in many solid tumors compared with normal tissues. This loss has been associated with increased tumor growth, impaired apoptosis, and poor prognosis, reflecting the classical tumor-suppressive and antioxidant functions of Sestrins. In contrast, SESN2 is overexpressed in melanoma and cutaneous squamous cell carcinoma and has been suggested to exert a pro-survival, AKT-supporting function, protecting transformed cells from ultraviolet- and stress-induced oxidative damage [23]. Studies using human skin-derived keratinocyte and fibroblast cultures have demonstrated that UVB exposure increases SESN2 expression and that this induction is abolished by p53 silencing, supporting a role for SESN2 in the UVB-induced stress response of skin cells [11]. Furthermore, upregulation of SESN2 following UVB exposure has been associated with the malignant transformation and survival of keratinocytes and melanocytes [24].

In the present study, dermal NRF2 expression decreased significantly following NB-UVB treatment, and this finding remained statistically significant after adjustment for multiple comparisons. No statistically significant changes were observed in epidermal NRF2 or epidermal and dermal SESN2 expression after adjustment. Although epidermal SESN2 expression increased numerically, this difference did not remain statistically significant following correction for multiple testing and should therefore be interpreted cautiously. No significant association was identified between cumulative NB-UVB dose and changes in SESN2 H-scores; however, this finding does not establish dose independence or clarify the biological mechanisms underlying the observed changes. Previous studies have shown that UVB exposure increases oxidative stress and may reduce NRF2 levels in epidermal keratinocytes, suggesting suppression of NRF2-mediated antioxidant defenses under UVB-induced stress [25]. UVB appears to be a weak activator of NRF2 signaling in the skin and may even suppress this pathway. In normal human epidermal keratinocytes and melanocytes, low-dose UVB exposure has been reported to markedly reduce NRF2 mRNA levels and the expression of NRF2 target genes, including heme oxygenase-1, glutathione S-transferase pi, and γ-glutamylcysteine synthetase [26].

In a study conducted in melanocytes, UVB was shown to induce SESN2 through p53- and AKT3-dependent pathways, whereas NRF2 did not appear to participate in this specific response. In contrast, NRF2 activation was reported to be associated with UVA exposure through a mitochondria-derived mechanism that was independent of SESN2. UVA exposure resulted in the accumulation of both NRF2 and SESN2; however, NRF2 silencing did not affect UVA-induced SESN2 upregulation. Furthermore, SESN2 ablation was associated with NRF2 accumulation and protection against UVA-induced oxidative stress [27]. UVA and UVB also differ substantially in their penetration through the layers of the skin. UVA penetrates more deeply and affects dermal structures, whereas UVB predominantly affects the epidermis [28]. Although previous experimental studies have demonstrated UVB-associated SESN2 induction, the numerical increase in epidermal SESN2 in our cohort did not remain statistically significant after adjustment for multiple comparisons. Our findings therefore do not provide sufficient evidence to support this mechanism. The decrease in dermal NRF2 may be related to reduced oxidative stress secondary to the loss of paracrine signaling from the diminished malignant T-cell population, together with the limited penetration of NB-UVB into the dermis. Neither epidermal SESN2 nor epidermal NRF2 expression showed a statistically significant change after adjustment for multiple comparisons. Accordingly, these findings do not establish whether NB-UVB elicits an NRF2-independent SESN2 response in epidermal keratinocytes.

Compared with other malignancies, the relationship between oxidative stress and MF remains relatively understudied. In lymphomas, NRF2 has been reported to act as a master regulator of antioxidant genes and to play an important role in both tumor cell survival and treatment resistance. Reducing NRF2 expression has also been associated with suppression of tumorigenesis and increased treatment responsiveness [29]. A recent study of cutaneous T-cell lymphoma (CTCL) demonstrated substantial oxidative stress in lesional MF skin, as evidenced by increased levels of 8-hydroxy-2′-deoxyguanosine and 4-hydroxy-2-nonenal, and showed that oxidative stress generated by malignant T cells could propagate to neighboring tissues through paracrine mechanisms. The same study reported decreased NRF2 gene expression and provided evidence that oxidative stress may act both as a driver of disease pathogenesis, by promoting malignant transformation and disruption of the tumor microenvironment, and as a potential therapeutic vulnerability that may facilitate selective elimination of malignant cells [12].

The cellular distribution of SESN2 and NRF2 immunoreactivity provides a framework for interpreting the observed treatment-associated changes. Epidermal immunoreactivity for both markers was localized to keratinocytes, whereas dermal SESN2 immunoreactivity was observed in lymphocytes. In contrast, dermal NRF2 immunoreactivity was distributed across cells morphologically consistent with fibroblasts, eosinophils, mast cells, lymphocytes, and histiocytes, suggesting contributions from multiple inflammatory and stromal populations. Consequently, the reduction in dermal NRF2 immunoreactivity following NB-UVB therapy may reflect alterations in the abundance or composition of NRF2-positive cell populations, changes in expression within individual cells, or a combination of these processes. Although this finding may be associated with remission-related remodeling of the dermal microenvironment, the present study design does not allow such changes to be distinguished from direct photobiological effects of NB-UVB. Furthermore, compartment-level immunohistochemical assessment precludes attribution of the observed reduction to individual cell populations or discrimination between the contributions of malignant and reactive lymphocytes. These interpretations therefore remain provisional and warrant further investigation using cell type-specific analyses and molecular validation.

Overall, clinical and histopathological remission following NB-UVB treatment in early-stage MF was associated with a significant reduction in dermal NRF2 expression after adjustment for multiple comparisons, whereas no statistically significant changes were observed in epidermal NRF2 or epidermal and dermal SESN2 expression. The present study cannot distinguish direct photobiological effects of NB-UVB from changes associated with remission, including alterations in the cellular composition of the tumor microenvironment. Further studies incorporating cell type-specific analyses and molecular validation are required to clarify the cellular sources and biological significance of the observed reduction in dermal NRF2 expression.

## 7. Limitations

This study has several limitations. First, its retrospective, single-center design and relatively small sample size may limit the generalizability of the findings. More importantly, the study design does not allow the direct biological effects of NB-UVB to be distinguished from biomarker changes associated with MF regression and histopathological remission. Second, the absence of untreated MF and healthy or non-lesional skin control groups precluded direct comparison of SESN2 and NRF2 expression with that in untreated lesional, non-lesional, or normal skin. Third, patients treated with different NB-UVB protocols or alternative treatment modalities were not included; therefore, potential treatment- or protocol-specific differences could not be assessed. Fourth, although the cellular distribution of SESN2 and NRF2 immunoreactivity was assessed morphologically, cell identities were not confirmed using lineage-specific double or multiplex staining. Furthermore, compartment-level scoring did not permit quantification of the contribution of individual cell populations or differentiation between malignant and reactive lymphocytes. Finally, oxidative stress was not measured directly, and SESN2- and NRF2-related signaling pathways were not evaluated at the molecular level. Thus, our findings are based on semiquantitative immunohistochemical evaluation rather than direct biochemical or molecular validation. Despite these limitations, the present findings provide a basis for future multicenter studies with larger patient cohorts and molecular validation to further elucidate the role of SESN2/NRF2-related signaling in the response of MF to NB-UVB treatment.

## Figures and Tables

**Table 1:** Demographic and clinical characteristics of the study population (*n* = 29).

Characteristic	Value
Patients, *n*	29
Age, years (mean ± SD)	52.0 ± 13.9
Sex, *n* (%)	
Female	17 (58.6)
Male	12 (41.4)
Disease stage, *n* (%)	
IA	9 (31.0)
IB	19 (65.5)
IIA	1 (3.4)
Time to histopathological remission weeks	
Mean ± SD	55.1 ± 26.8
Median (range)	52 (23–135)
Cumulative total UVB dose mJ/cm^2^	
Mean ± SD	189,206.9 ± 125,933.2
Median (range)	117,000 (6500–440,000)

SD, standard deviation; IA/IB/IIA, mycosis fungoides clinical stage; mJ/cm^2^, millijoules per square centimeter.

**Table 2:** Comparison of epidermal and dermal SESN2 and NRF2 expression before and after NB-UVB phototherapy.

Marker/Compartment	Measure	Before Treatment Median (IQR)	After Treatment Median (IQR)	*p*	Adjusted *p* (Within Measure) ^a^	Adjusted *p* (All Tests) ^b^	r
SESN2/Epidermis	Intensity (0–3)	1 (1–2)	2 (1–3)	0.058	0.174	0.406	0.35
	Positive cells (%)	30 (10–40)	40 (20–50)	0.047	0.141	0.375	0.37
	H-score (0–300)	30 (10–60)	80 (20–120)	0.030	0.091	0.272	0.40
SESN2/Dermis	Intensity (0–3)	1 (1–2)	1 (1–2)	0.734	1.000	1.000	0.06
	Positive cells (%)	30 (5–70)	20 (5–70)	0.791	1.000	1.000	0.05
	H-score (0–300)	30 (5–140)	40 (5–160)	0.820	1.000	1.000	0.04
NRF2/Epidermis	Intensity (0–3)	2 (1–3)	2 (2–2)	0.651	1.000	1.000	0.08
	Positive cells (%)	40 (30–60)	40 (30–60)	0.858	1.000	1.000	0.03
	H-score (0–300)	90 (30–180)	80 (50–120)	0.657	1.000	1.000	0.08
NRF2/Dermis	Intensity (0–3)	2 (2–3)	0 (0–2)	<0.001 *	0.001 *	0.004 *	0.67
	Positive cells (%)	40 (10–60)	0 (0–20)	0.002 *	0.006 *	0.018 *	0.59
	H-score (0–300)	80 (30–120)	0 (0–60)	0.002 *	0.007 *	0.018 *	0.58

Data are presented as median (interquartile range, IQR). Pre- and post-treatment values were compared using the Wilcoxon signed-rank test. ^a^ Holm-adjusted for the four comparisons within each measure (Sestrin and Nrf2 in the epidermis and dermis). ^b^ Holm-adjusted across all 12 comparisons. r, effect size: 0.1 small, 0.3 medium, ≥0.5 large. * *p* < 0.05.

**Table 3:** Correlation between cumulative UVB dose and the magnitude of change in SESN2 and NRF2 parameters (*n* = 29).

Parameter	Spearman ρ	*p*
Epidermal SESN2 H-score	−0.16	0.418
Dermal SESN2 H-score	0.08	0.665
Epidermal NRF2 H-score	−0.04	0.853
Dermal NRF2 H-score	0.20	0.304

ρ, Spearman correlation coefficient between the individual change score (post − pre) for each parameter and the cumulative total mJ/cm^2^ dose received at treatment completion; values in bold indicate statistical significance (*p* < 0.05).

**Table 4:** Correlation between epidermal and dermal SESN2 and NRF2 expression and time to histopathological remission (*n* = 29).

Parameter	Baseline ^a^		Post-Treatment ^b^		Change ^c^	
	ρ	*p*	ρ	*p*	ρ	*p*
Epidermal SESN2 H-score	0.01	0.965	−0.07	0.719	−0.09	0.630
Dermal SESN2 H-score	−0.05	0.811	−0.04	0.843	−0.07	0.736
Epidermal NRF2 H-score	−0.05	0.790	0.11	0.575	0.06	0.768
Dermal NRF2 H-score	−0.29	0.129	−0.02	0.923	0.12	0.545

ρ, Spearman correlation coefficient with time to histopathological remission (weeks); ^a^ Pre-treatment (baseline) parameter value. ^b^ Post-treatment parameter value; ^c^ Magnitude of change (post-treatment minus pre-treatment value).

## Data Availability

The data used in this study were obtained from retrospective archive records. Due to privacy and ethical restrictions, the datasets are not publicly available but are available from the corresponding author upon reasonable request.
